# Supplementary figures and images for: Rating enrichment items by female group-housed laboratory mice in multiple binary choice tests using an RFID-based tracking system
Source: PLoS One. 2023 Jan 19;18(1):e0278709. doi: 10.1371/journal.pone.0278709 (PMC9851564; doi:10.1371/journal.pone.0278709)

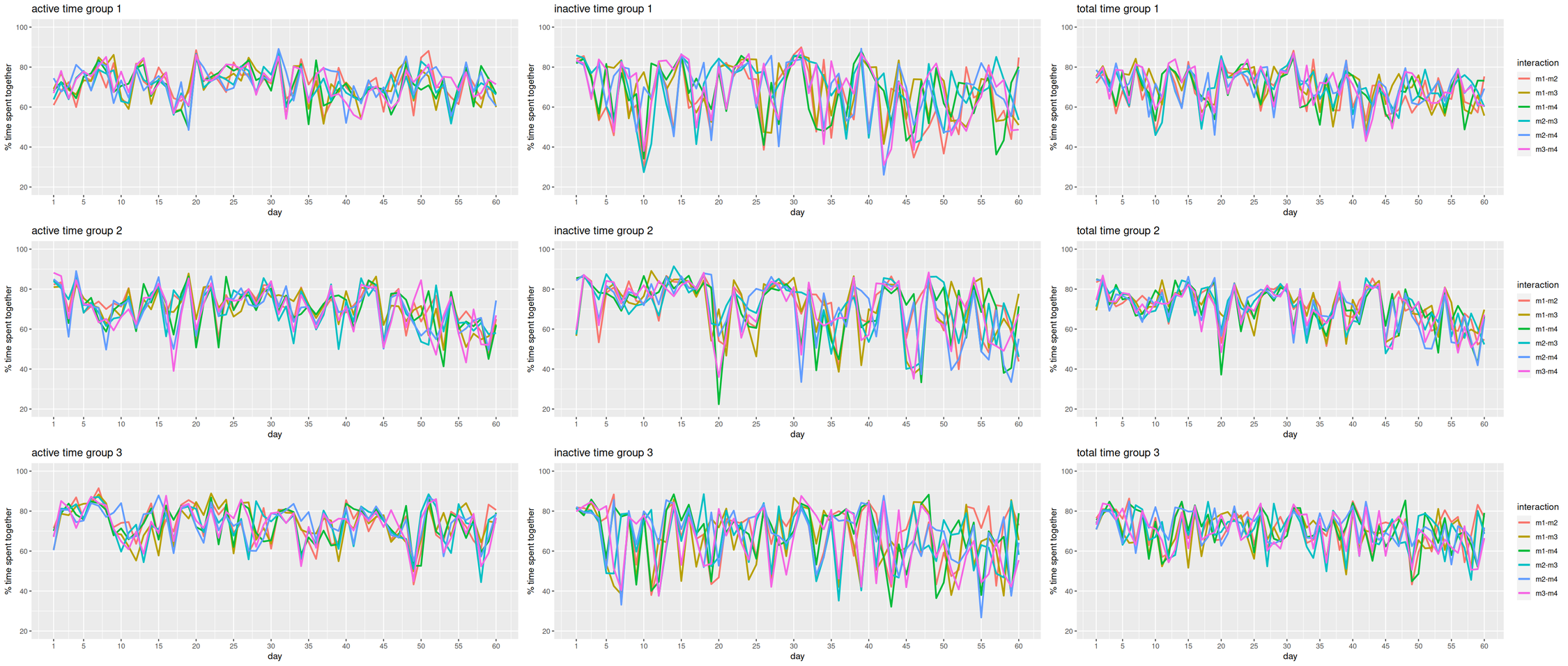

Supplement: S1 Fig — Mice were displayed as couples (m1-m2: mouse 1 and mouse 2, m1-m3: mouse 1 and mouse 3, m1-m4: mouse 1 and mouse 4, m2-m3: mouse 2 and mouse 3, m2-m4: mouse 2 and mouse 4, m3-m4: mouse 3 and mouse 4). (TIF) [file pone.0278709.s001.tif]
