# Supplementary material for: Rating enrichment items by female group-housed laboratory mice in multiple binary choice tests using an RFID-based tracking system
Source: PLoS One. 2023 Jan 19;18(1):e0278709. doi: 10.1371/journal.pone.0278709 (PMC9851564; doi:10.1371/journal.pone.0278709)
Supplement: S1 Table — (PDF) [file pone.0278709.s002.pdf]

	compiled_studies	subject ID	group	option A	option B	quantity A in %	quantity B in %
1	structural	mouse1	1	rope	mouseswing	37.37847	62.62153
2	structural	mouse1	1	clip_papertube	rope	42.53819	57.46181
3	structural	mouse1	1	mouseswing	clip_papertube	43.76157	56.23843
4	structural	mouse1	1	clip_papertube	second_plane	39.96181	60.03819
5	structural	mouse1	1	second_plane	rope	18.83333	81.16667
6	structural	mouse1	1	clip_plastictube	rope	32.69907	67.30093
7	structural	mouse1	1	clip_plastictube	second_plane	31.71528	68.28472
8	structural	mouse1	1	clip_plastictube	clip_papertube	44.65972	55.34028
9	structural	mouse1	1	clip_plastictube	mouseswing	45.68866	54.31134
10	structural	mouse1	1	second_plane	mouseswing	64.51736	35.48264
11	foraging	mouse1	1	treatball	latticeball	69.21181	30.78819
12	foraging	mouse1	1	tube_stones	flappuzzle	55.74074	44.25926
13	foraging	mouse1	1	flappuzzle	treatball	63.89931	36.10069
14	foraging	mouse1	1	tube_stones	latticeball	32.44097	67.55903
15	foraging	mouse1	1	tube_stones	slidingpuzzle	62.30556	37.69444
16	foraging	mouse1	1	tube_stones	treatball	70.48264	29.51736
17	foraging	mouse1	1	latticeball	slidingpuzzle	74.81250	25.18750
18	foraging	mouse1	1	treatball	slidingpuzzle	42.19676	57.80324
19	foraging	mouse1	1	flappuzzle	slidingpuzzle	60.18634	39.81366
20	foraging	mouse1	1	latticeball	flappuzzle	62.20486	37.79514
21	housing	mouse1	1	floorhouse	woodenangle	52.60301	47.39699
22	housing	mouse1	1	woodenangle_with_hole	houseball	43.51042	56.48958
23	housing	mouse1	1	woodenangle_with_hole	woodenangle	40.73148	59.26852
24	housing	mouse1	1	woodenangle	paperhouse	64.19907	35.80093
25	housing	mouse1	1	paperhouse	floorhouse	50.16551	49.83449
26	housing	mouse1	1	floorhouse	woodenangle_with_hole	29.82407	70.17593
27	housing	mouse1	1	paperhouse	woodenangle_with_hole	44.48148	55.51852
28	housing	mouse1	1	houseball	floorhouse	41.59375	58.40625
29	housing	mouse1	1	woodenangle	houseball	41.87616	58.12384
30	housing	mouse1	1	houseball	paperhouse	42.83681	57.16319
31	structural	mouse2	1	rope	mouseswing	30.39815	69.60185
32	structural	mouse2	1	clip_papertube	rope	34.74651	65.25349
33	structural	mouse2	1	mouseswing	clip_papertube	42.95370	57.04630
34	structural	mouse2	1	clip_papertube	second_plane	50.19792	49.80208
35	structural	mouse2	1	second_plane	rope	25.73032	74.26968
36	structural	mouse2	1	clip_plastictube	rope	42.37963	57.62037
37	structural	mouse2	1	clip_plastictube	second_plane	39.39815	60.60185
38	structural	mouse2	1	clip_plastictube	clip_papertube	41.74537	58.25463
39	structural	mouse2	1	clip_plastictube	mouseswing	51.53241	48.46759
40	structural	mouse2	1	second_plane	mouseswing	76.08333	23.91667
41	foraging	mouse2	1	treatball	latticeball	67.89815	32.10185
42	foraging	mouse2	1	tube_stones	flappuzzle	54.89236	45.10764
43	foraging	mouse2	1	flappuzzle	treatball	60.88657	39.11343
44	foraging	mouse2	1	tube_stones	latticeball	32.47569	67.52431
45	foraging	mouse2	1	tube_stones	slidingpuzzle	63.56597	36.43403
46	foraging	mouse2	1	tube_stones	treatball	69.72222	30.27778
47	foraging	mouse2	1	latticeball	slidingpuzzle	73.23148	26.76852
48	foraging	mouse2	1	treatball	slidingpuzzle	47.52894	52.47106
49	foraging	mouse2	1	flappuzzle	slidingpuzzle	52.90046	47.09954
50	foraging	mouse2	1	latticeball	flappuzzle	58.04051	41.95949
51	housing	mouse2	1	floorhouse	woodenangle	62.00463	37.99537
52	housing	mouse2	1	woodenangle_with_hole	houseball	38.63194	61.36806
53	housing	mouse2	1	woodenangle_with_hole	woodenangle	54.37037	45.62963
54	housing	mouse2	1	woodenangle	paperhouse	52.95139	47.04861
55	housing	mouse2	1	paperhouse	floorhouse	50.99769	49.00231
56	housing	mouse2	1	floorhouse	woodenangle_with_hole	22.17477	77.82523
57	housing	mouse2	1	paperhouse	woodenangle_with_hole	56.12037	43.87963
58	housing	mouse2	1	houseball	floorhouse	33.41435	66.58565
59	housing	mouse2	1	woodenangle	houseball	54.05556	45.94444
60	housing	mouse2	1	houseball	paperhouse	42.45949	57.54051
61	structural	mouse3	1	rope	mouseswing	37.67361	62.32639
62	structural	mouse3	1	clip_papertube	rope	47.57993	52.42007
63	structural	mouse3	1	mouseswing	clip_papertube	44.83102	55.16898
64	structural	mouse3	1	clip_papertube	second_plane	43.76389	56.23611
65	structural	mouse3	1	second_plane	rope	21.89352	78.10648
66	structural	mouse3	1	clip_plastictube	rope	41.03009	58.96991
67	structural	mouse3	1	clip_plastictube	second_plane	34.54167	65.45833
68	structural	mouse3	1	clip_plastictube	clip_papertube	50.08565	49.91435
69	structural	mouse3	1	clip_plastictube	mouseswing	55.94792	44.05208
70	structural	mouse3	1	second_plane	mouseswing	62.40046	37.59954
71	foraging	mouse3	1	treatball	latticeball	68.70023	31.29977
72	foraging	mouse3	1	tube_stones	flappuzzle	57.78472	42.21528
73	foraging	mouse3	1	flappuzzle	treatball	66.43519	33.56481
74	foraging	mouse3	1	tube_stones	latticeball	33.67130	66.32870
75	foraging	mouse3	1	tube_stones	slidingpuzzle	68.47454	31.52546
76	foraging	mouse3	1	tube_stones	treatball	61.23495	38.76505
77	foraging	mouse3	1	latticeball	slidingpuzzle	74.27315	25.72685
78	foraging	mouse3	1	treatball	slidingpuzzle	51.17361	48.82639
79	foraging	mouse3	1	flappuzzle	slidingpuzzle	69.53125	30.46875
80	foraging	mouse3	1	latticeball	flappuzzle	63.13194	36.86806
81	housing	mouse3	1	floorhouse	woodenangle	54.68519	45.31481
82	housing	mouse3	1	woodenangle_with_hole	houseball	47.45833	52.54167
83	housing	mouse3	1	woodenangle_with_hole	woodenangle	33.55903	66.44097
84	housing	mouse3	1	woodenangle	paperhouse	65.91667	34.08333
85	housing	mouse3	1	paperhouse	floorhouse	39.38889	60.61111
86	housing	mouse3	1	floorhouse	woodenangle_with_hole	24.82986	75.17014
87	housing	mouse3	1	paperhouse	woodenangle_with_hole	60.11574	39.88426
88	housing	mouse3	1	houseball	floorhouse	30.88889	69.11111
89	housing	mouse3	1	woodenangle	houseball	55.49306	44.50694
90	housing	mouse3	1	houseball	paperhouse	40.42245	59.57755
91	structural	mouse4	1	rope	mouseswing	34.97685	65.02315
92	structural	mouse4	1	clip_papertube	rope	39.96412	60.03588
93	structural	mouse4	1	mouseswing	clip_papertube	47.22454	52.77546
94	structural	mouse4	1	clip_papertube	second_plane	46.06019	53.93981
95	structural	mouse4	1	second_plane	rope	26.85185	73.14815
96	structural	mouse4	1	clip_plastictube	rope	41.07060	58.92940
97	structural	mouse4	1	clip_plastictube	second_plane	42.93171	57.06829
98	structural	mouse4	1	clip_plastictube	clip_papertube	45.48725	54.51275
99	structural	mouse4	1	clip_plastictube	mouseswing	44.03125	55.96875
100	structural	mouse4	1	second_plane	mouseswing	61.58218	38.41782
101	foraging	mouse4	1	treatball	latticeball	66.14699	33.85301
102	foraging	mouse4	1	tube_stones	flappuzzle	55.46991	44.53009
103	foraging	mouse4	1	flappuzzle	treatball	59.14236	40.85764
104	foraging	mouse4	1	tube_stones	latticeball	30.31250	69.68750
105	foraging	mouse4	1	tube_stones	slidingpuzzle	74.86806	25.13194
106	foraging	mouse4	1	tube_stones	treatball	71.36227	28.63773
107	foraging	mouse4	1	latticeball	slidingpuzzle	76.32755	23.67245
108	foraging	mouse4	1	treatball	slidingpuzzle	38.00694	61.99306
109	foraging	mouse4	1	flappuzzle	slidingpuzzle	59.82870	40.17130
110	foraging	mouse4	1	latticeball	flappuzzle	60.98495	39.01505
111	housing	mouse4	1	floorhouse	woodenangle	62.93634	37.06366
112	housing	mouse4	1	woodenangle_with_hole	houseball	40.78588	59.21412
113	housing	mouse4	1	woodenangle_with_hole	woodenangle	50.13657	49.86343
114	housing	mouse4	1	woodenangle	paperhouse	59.90625	40.09375
115	housing	mouse4	1	paperhouse	floorhouse	40.65625	59.34375
116	housing	mouse4	1	floorhouse	woodenangle_with_hole	22.62269	77.37731
117	housing	mouse4	1	paperhouse	woodenangle_with_hole	63.99306	36.00694
118	housing	mouse4	1	houseball	floorhouse	36.58102	63.41898
119	housing	mouse4	1	woodenangle	houseball	50.00579	49.99421
120	housing	mouse4	1	houseball	paperhouse	42.87037	57.12963
121	structural	mouse5	2	rope	mouseswing	47.82639	52.17361
122	structural	mouse5	2	clip_papertube	rope	63.23727	36.76273
123	structural	mouse5	2	mouseswing	clip_papertube	49.21296	50.78704
124	structural	mouse5	2	clip_papertube	second_plane	57.53935	42.46065
125	structural	mouse5	2	second_plane	rope	47.65972	52.34028
126	structural	mouse5	2	clip_plastictube	rope	52.02199	47.97801
127	structural	mouse5	2	clip_plastictube	second_plane	61.15046	38.84954
128	structural	mouse5	2	clip_plastictube	clip_papertube	43.53935	56.46065
129	structural	mouse5	2	clip_plastictube	mouseswing	52.63542	47.36458
130	structural	mouse5	2	second_plane	mouseswing	73.68403	26.31597
131	foraging	mouse5	2	treatball	latticeball	39.45255	60.54745
132	foraging	mouse5	2	tube_stones	flappuzzle	60.76505	39.23495
133	foraging	mouse5	2	flappuzzle	treatball	57.26505	42.73495
134	foraging	mouse5	2	tube_stones	latticeball	15.35880	84.64120
135	foraging	mouse5	2	tube_stones	slidingpuzzle	53.08681	46.91319
136	foraging	mouse5	2	tube_stones	treatball	82.44329	17.55671
137	foraging	mouse5	2	latticeball	slidingpuzzle	71.25926	28.74074
138	foraging	mouse5	2	treatball	slidingpuzzle	45.72338	54.27662
139	foraging	mouse5	2	flappuzzle	slidingpuzzle	75.72685	24.27315
140	foraging	mouse5	2	latticeball	flappuzzle	37.30787	62.69213
141	housing	mouse5	2	floorhouse	woodenangle	75.25810	24.74190
142	housing	mouse5	2	woodenangle_with_hole	houseball	47.94097	52.05903
143	housing	mouse5	2	woodenangle_with_hole	woodenangle	53.56128	46.43872
144	housing	mouse5	2	woodenangle	paperhouse	21.15509	78.84491
145	housing	mouse5	2	paperhouse	floorhouse	53.89583	46.10417
146	housing	mouse5	2	floorhouse	woodenangle_with_hole	62.73465	37.26535
147	housing	mouse5	2	paperhouse	woodenangle_with_hole	57.30671	42.69329
148	housing	mouse5	2	houseball	floorhouse	51.81134	48.18866
149	housing	mouse5	2	woodenangle	houseball	56.65278	43.34722
150	housing	mouse5	2	houseball	paperhouse	47.37384	52.62616
151	structural	mouse6	2	rope	mouseswing	47.11805	52.88195
152	structural	mouse6	2	clip_papertube	rope	57.66088	42.33912
153	structural	mouse6	2	mouseswing	clip_papertube	51.10532	48.89468
154	structural	mouse6	2	clip_papertube	second_plane	53.02315	46.97685
155	structural	mouse6	2	second_plane	rope	46.29514	53.70486
156	structural	mouse6	2	clip_plastictube	rope	50.44792	49.55208
157	structural	mouse6	2	clip_plastictube	second_plane	63.50463	36.49537
158	structural	mouse6	2	clip_plastictube	clip_papertube	37.42477	62.57523
159	structural	mouse6	2	clip_plastictube	mouseswing	44.40046	55.59954
160	structural	mouse6	2	second_plane	mouseswing	78.95255	21.04745
161	foraging	mouse6	2	treatball	latticeball	38.99190	61.00810
162	foraging	mouse6	2	tube_stones	flappuzzle	62.79630	37.20370
163	foraging	mouse6	2	flappuzzle	treatball	48.10648	51.89352
164	foraging	mouse6	2	tube_stones	latticeball	21.28241	78.71759
165	foraging	mouse6	2	tube_stones	slidingpuzzle	54.98032	45.01968
166	foraging	mouse6	2	tube_stones	treatball	77.06713	22.93287
167	foraging	mouse6	2	latticeball	slidingpuzzle	63.66782	36.33218
168	foraging	mouse6	2	treatball	slidingpuzzle	46.15046	53.84954
169	foraging	mouse6	2	flappuzzle	slidingpuzzle	73.52083	26.47917
170	foraging	mouse6	2	latticeball	flappuzzle	41.42593	58.57407
171	housing	mouse6	2	floorhouse	woodenangle	73.45949	26.54051
172	housing	mouse6	2	woodenangle_with_hole	houseball	47.13079	52.86921
173	housing	mouse6	2	woodenangle_with_hole	woodenangle	64.20949	35.79051
174	housing	mouse6	2	woodenangle	paperhouse	18.79398	81.20602
175	housing	mouse6	2	paperhouse	floorhouse	56.63657	43.36343
176	housing	mouse6	2	floorhouse	woodenangle_with_hole	58.90741	41.09259
177	housing	mouse6	2	paperhouse	woodenangle_with_hole	74.27083	25.72917
178	housing	mouse6	2	houseball	floorhouse	53.92361	46.07639
179	housing	mouse6	2	woodenangle	houseball	50.12384	49.87616
180	housing	mouse6	2	houseball	paperhouse	34.51968	65.48032
181	structural	mouse7	2	rope	mouseswing	50.89468	49.10532
182	structural	mouse7	2	clip_papertube	rope	52.58218	47.41782
183	structural	mouse7	2	mouseswing	clip_papertube	54.98727	45.01273
184	structural	mouse7	2	clip_papertube	second_plane	48.79745	51.20255
185	structural	mouse7	2	second_plane	rope	55.51852	44.48148
186	structural	mouse7	2	clip_plastictube	rope	39.83796	60.16204
187	structural	mouse7	2	clip_plastictube	second_plane	59.93831	40.06169
188	structural	mouse7	2	clip_plastictube	clip_papertube	46.51620	53.48380
189	structural	mouse7	2	clip_plastictube	mouseswing	53.00231	46.99769
190	structural	mouse7	2	second_plane	mouseswing	81.95139	18.04861
191	foraging	mouse7	2	treatball	latticeball	39.36458	60.63542
192	foraging	mouse7	2	tube_stones	flappuzzle	74.07176	25.92824
193	foraging	mouse7	2	flappuzzle	treatball	51.71065	48.28935
194	foraging	mouse7	2	tube_stones	latticeball	19.22685	80.77315
195	foraging	mouse7	2	tube_stones	slidingpuzzle	46.83333	53.16667
196	foraging	mouse7	2	tube_stones	treatball	76.83218	23.16782
197	foraging	mouse7	2	latticeball	slidingpuzzle	75.52894	24.47106
198	foraging	mouse7	2	treatball	slidingpuzzle	44.25463	55.74537
199	foraging	mouse7	2	flappuzzle	slidingpuzzle	72.08102	27.91898
200	foraging	mouse7	2	latticeball	flappuzzle	49.49074	50.50926
201	housing	mouse7	2	floorhouse	woodenangle	75.54167	24.45833
202	housing	mouse7	2	woodenangle_with_hole	houseball	51.59144	48.40856
203	housing	mouse7	2	woodenangle_with_hole	woodenangle	51.04051	48.95949
204	housing	mouse7	2	woodenangle	paperhouse	27.67130	72.32870
205	housing	mouse7	2	paperhouse	floorhouse	57.43287	42.56713
206	housing	mouse7	2	floorhouse	woodenangle_with_hole	47.01505	52.98495
207	housing	mouse7	2	paperhouse	woodenangle_with_hole	71.90046	28.09954
208	housing	mouse7	2	houseball	floorhouse	45.64699	54.35301
209	housing	mouse7	2	woodenangle	houseball	51.89699	48.10301
210	housing	mouse7	2	houseball	paperhouse	32.43634	67.56366
211	structural	mouse8	2	rope	mouseswing	50.53125	49.46875
212	structural	mouse8	2	clip_papertube	rope	62.83102	37.16898
213	structural	mouse8	2	mouseswing	clip_papertube	48.02083	51.97917
214	structural	mouse8	2	clip_papertube	second_plane	56.42824	43.57176
215	structural	mouse8	2	second_plane	rope	50.94676	49.05324
216	structural	mouse8	2	clip_plastictube	rope	44.16551	55.83449
217	structural	mouse8	2	clip_plastictube	second_plane	65.34375	34.65625
218	structural	mouse8	2	clip_plastictube	clip_papertube	46.84283	53.15717
219	structural	mouse8	2	clip_plastictube	mouseswing	64.04977	35.95023
220	structural	mouse8	2	second_plane	mouseswing	71.29514	28.70486
221	foraging	mouse8	2	treatball	latticeball	37.65278	62.34722
222	foraging	mouse8	2	tube_stones	flappuzzle	60.89352	39.10648
223	foraging	mouse8	2	flappuzzle	treatball	52.94792	47.05208
224	foraging	mouse8	2	tube_stones	latticeball	15.72685	84.27315
225	foraging	mouse8	2	tube_stones	slidingpuzzle	50.87847	49.12153
226	foraging	mouse8	2	tube_stones	treatball	75.66551	24.33449
227	foraging	mouse8	2	latticeball	slidingpuzzle	76.41204	23.58796
228	foraging	mouse8	2	treatball	slidingpuzzle	45.96991	54.03009
229	foraging	mouse8	2	flappuzzle	slidingpuzzle	75.30671	24.69329
230	foraging	mouse8	2	latticeball	flappuzzle	41.45370	58.54630
231	housing	mouse8	2	floorhouse	woodenangle	67.55093	32.44907
232	housing	mouse8	2	woodenangle_with_hole	houseball	49.41435	50.58565
233	housing	mouse8	2	woodenangle_with_hole	woodenangle	60.98843	39.01157
234	housing	mouse8	2	woodenangle	paperhouse	13.03009	86.96991
235	housing	mouse8	2	paperhouse	floorhouse	56.46181	43.53819
236	housing	mouse8	2	floorhouse	woodenangle_with_hole	67.16667	32.83333
237	housing	mouse8	2	paperhouse	woodenangle_with_hole	79.23495	20.76505
238	housing	mouse8	2	houseball	floorhouse	48.38542	51.61458
239	housing	mouse8	2	woodenangle	houseball	58.72106	41.27894
240	housing	mouse8	2	houseball	paperhouse	34.91898	65.08102
241	structural	mouse9	3	rope	mouseswing	36.46528	63.53472
242	structural	mouse9	3	clip_papertube	rope	52.30440	47.69560
243	structural	mouse9	3	mouseswing	clip_papertube	14.52778	85.47222
244	structural	mouse9	3	clip_papertube	second_plane	45.38426	54.61574
245	structural	mouse9	3	second_plane	rope	62.50231	37.49769
246	structural	mouse9	3	clip_plastictube	rope	51.32176	48.67824
247	structural	mouse9	3	clip_plastictube	second_plane	40.38542	59.61458
248	structural	mouse9	3	clip_plastictube	clip_papertube	76.43056	23.56944
249	structural	mouse9	3	clip_plastictube	mouseswing	76.31366	23.68634
250	structural	mouse9	3	second_plane	mouseswing	31.59491	68.40509
251	foraging	mouse9	3	treatball	latticeball	20.26389	79.73611
252	foraging	mouse9	3	tube_stones	flappuzzle	24.31366	75.68634
253	foraging	mouse9	3	flappuzzle	treatball	29.41898	70.58102
254	foraging	mouse9	3	tube_stones	latticeball	41.42014	58.57986
255	foraging	mouse9	3	tube_stones	slidingpuzzle	34.52315	65.47685
256	foraging	mouse9	3	tube_stones	treatball	53.84028	46.15972
257	foraging	mouse9	3	latticeball	slidingpuzzle	71.60532	28.39468
258	foraging	mouse9	3	treatball	slidingpuzzle	59.21181	40.78819
259	foraging	mouse9	3	flappuzzle	slidingpuzzle	58.11806	41.88194
260	foraging	mouse9	3	latticeball	flappuzzle	64.29861	35.70139
261	housing	mouse9	3	floorhouse	woodenangle	56.47106	43.52894
262	housing	mouse9	3	woodenangle_with_hole	houseball	52.65046	47.34954
263	housing	mouse9	3	woodenangle_with_hole	woodenangle	41.14699	58.85301
264	housing	mouse9	3	woodenangle	paperhouse	67.24190	32.75810
265	housing	mouse9	3	paperhouse	floorhouse	50.03588	49.96412
266	housing	mouse9	3	floorhouse	woodenangle_with_hole	24.47801	75.52199
267	housing	mouse9	3	paperhouse	woodenangle_with_hole	60.87153	39.12847
268	housing	mouse9	3	houseball	floorhouse	19.31250	80.68750
269	housing	mouse9	3	woodenangle	houseball	65.24653	34.75347
270	housing	mouse9	3	houseball	paperhouse	50.49421	49.50579
271	structural	mouse10	3	rope	mouseswing	37.12384	62.87616
272	structural	mouse10	3	clip_papertube	rope	49.49306	50.50694
273	structural	mouse10	3	mouseswing	clip_papertube	18.30440	81.69560
274	structural	mouse10	3	clip_papertube	second_plane	46.23032	53.76968
275	structural	mouse10	3	second_plane	rope	54.05324	45.94676
276	structural	mouse10	3	clip_plastictube	rope	53.90741	46.09259
277	structural	mouse10	3	clip_plastictube	second_plane	43.30093	56.69907
278	structural	mouse10	3	clip_plastictube	clip_papertube	65.58796	34.41204
279	structural	mouse10	3	clip_plastictube	mouseswing	65.58912	34.41088
280	structural	mouse10	3	second_plane	mouseswing	35.40162	64.59838
281	foraging	mouse10	3	treatball	latticeball	20.19213	79.80787
282	foraging	mouse10	3	tube_stones	flappuzzle	28.73958	71.26042
283	foraging	mouse10	3	flappuzzle	treatball	26.96759	73.03241
284	foraging	mouse10	3	tube_stones	latticeball	37.57755	62.42245
285	foraging	mouse10	3	tube_stones	slidingpuzzle	29.19444	70.80556
286	foraging	mouse10	3	tube_stones	treatball	47.97338	52.02662
287	foraging	mouse10	3	latticeball	slidingpuzzle	77.50347	22.49653
288	foraging	mouse10	3	treatball	slidingpuzzle	65.11227	34.88773
289	foraging	mouse10	3	flappuzzle	slidingpuzzle	64.39120	35.60880
290	foraging	mouse10	3	latticeball	flappuzzle	62.87731	37.12269
291	housing	mouse10	3	floorhouse	woodenangle	53.08565	46.91435
292	housing	mouse10	3	woodenangle_with_hole	houseball	49.52083	50.47917
293	housing	mouse10	3	woodenangle_with_hole	woodenangle	43.97917	56.02083
294	housing	mouse10	3	woodenangle	paperhouse	70.53241	29.46759
295	housing	mouse10	3	paperhouse	floorhouse	39.18403	60.81597
296	housing	mouse10	3	floorhouse	woodenangle_with_hole	29.67593	70.32407
297	housing	mouse10	3	paperhouse	woodenangle_with_hole	57.27199	42.72801
298	housing	mouse10	3	houseball	floorhouse	21.59375	78.40625
299	housing	mouse10	3	woodenangle	houseball	51.00810	48.99190
300	housing	mouse10	3	houseball	paperhouse	55.47685	44.52315
301	structural	mouse11	3	rope	mouseswing	38.60069	61.39931
302	structural	mouse11	3	clip_papertube	rope	49.66204	50.33796
303	structural	mouse11	3	mouseswing	clip_papertube	14.94907	85.05093
304	structural	mouse11	3	clip_papertube	second_plane	48.46181	51.53819
305	structural	mouse11	3	second_plane	rope	53.80440	46.19560
306	structural	mouse11	3	clip_plastictube	rope	48.10995	51.89005
307	structural	mouse11	3	clip_plastictube	second_plane	40.96991	59.03009
308	structural	mouse11	3	clip_plastictube	clip_papertube	61.90162	38.09838
309	structural	mouse11	3	clip_plastictube	mouseswing	79.20023	20.79977
310	structural	mouse11	3	second_plane	mouseswing	30.82639	69.17361
311	foraging	mouse11	3	treatball	latticeball	17.81366	82.18634
312	foraging	mouse11	3	tube_stones	flappuzzle	24.53704	75.46296
313	foraging	mouse11	3	flappuzzle	treatball	20.84606	79.15394
314	foraging	mouse11	3	tube_stones	latticeball	29.00000	71.00000
315	foraging	mouse11	3	tube_stones	slidingpuzzle	32.15625	67.84375
316	foraging	mouse11	3	tube_stones	treatball	46.59838	53.40162
317	foraging	mouse11	3	latticeball	slidingpuzzle	67.19329	32.80671
318	foraging	mouse11	3	treatball	slidingpuzzle	57.19329	42.80671
319	foraging	mouse11	3	flappuzzle	slidingpuzzle	63.42477	36.57523
320	foraging	mouse11	3	latticeball	flappuzzle	56.48495	43.51505
321	housing	mouse11	3	floorhouse	woodenangle	59.91319	40.08681
322	housing	mouse11	3	woodenangle_with_hole	houseball	46.48727	53.51273
323	housing	mouse11	3	woodenangle_with_hole	woodenangle	37.00926	62.99074
324	housing	mouse11	3	woodenangle	paperhouse	60.38657	39.61343
325	housing	mouse11	3	paperhouse	floorhouse	45.04977	54.95023
326	housing	mouse11	3	floorhouse	woodenangle_with_hole	31.29977	68.70023
327	housing	mouse11	3	paperhouse	woodenangle_with_hole	64.36343	35.63657
328	housing	mouse11	3	houseball	floorhouse	21.35995	78.64005
329	housing	mouse11	3	woodenangle	houseball	66.57060	33.42940
330	housing	mouse11	3	houseball	paperhouse	49.53935	50.46065
331	structural	mouse12	3	rope	mouseswing	41.87963	58.12037
332	structural	mouse12	3	clip_papertube	rope	44.15509	55.84491
333	structural	mouse12	3	mouseswing	clip_papertube	15.82639	84.17361
334	structural	mouse12	3	clip_papertube	second_plane	44.44907	55.55093
335	structural	mouse12	3	second_plane	rope	49.78009	50.21991
336	structural	mouse12	3	clip_plastictube	rope	55.64005	44.35995
337	structural	mouse12	3	clip_plastictube	second_plane	47.29514	52.70486
338	structural	mouse12	3	clip_plastictube	clip_papertube	69.86921	30.13079
339	structural	mouse12	3	clip_plastictube	mouseswing	82.39699	17.60301
340	structural	mouse12	3	second_plane	mouseswing	32.25000	67.75000
341	foraging	mouse12	3	treatball	latticeball	23.28935	76.71065
342	foraging	mouse12	3	tube_stones	flappuzzle	23.37153	76.62847
343	foraging	mouse12	3	flappuzzle	treatball	18.13773	81.86227
344	foraging	mouse12	3	tube_stones	latticeball	38.01042	61.98958
345	foraging	mouse12	3	tube_stones	slidingpuzzle	27.16898	72.83102
346	foraging	mouse12	3	tube_stones	treatball	52.94213	47.05787
347	foraging	mouse12	3	latticeball	slidingpuzzle	74.70370	25.29630
348	foraging	mouse12	3	treatball	slidingpuzzle	60.12731	39.87269
349	foraging	mouse12	3	flappuzzle	slidingpuzzle	68.39815	31.60185
350	foraging	mouse12	3	latticeball	flappuzzle	59.93171	40.06829
351	housing	mouse12	3	floorhouse	woodenangle	57.91088	42.08912
352	housing	mouse12	3	woodenangle_with_hole	houseball	51.03704	48.96296
353	housing	mouse12	3	woodenangle_with_hole	woodenangle	39.43287	60.56713
354	housing	mouse12	3	woodenangle	paperhouse	66.76620	33.23380
355	housing	mouse12	3	paperhouse	floorhouse	32.18056	67.81944
356	housing	mouse12	3	floorhouse	woodenangle_with_hole	33.64931	66.35069
357	housing	mouse12	3	paperhouse	woodenangle_with_hole	55.25231	44.74769
358	housing	mouse12	3	houseball	floorhouse	19.75000	80.25000
359	housing	mouse12	3	woodenangle	houseball	69.05556	30.94444
360	housing	mouse12	3	houseball	paperhouse	61.96412	38.03588
