# Supplementary material for: Rating enrichment items by female group-housed laboratory mice in multiple binary choice tests using an RFID-based tracking system
Source: PLoS One. 2023 Jan 19;18(1):e0278709. doi: 10.1371/journal.pone.0278709 (PMC9851564; doi:10.1371/journal.pone.0278709)
Supplement: Attachment — Submitted filename: Rewiever Kommentare.pdf [file pone.0278709.s005.pdf]

Reviewers' comments:

Reviewer's Responses to Questions

Comments to the Author

1. Is the manuscript technically sound, and do the data support the conclusions?

The manuscript must describe a technically sound piece of scientific research with data that supports the conclusions. Experiments must have been conducted rigorously, with appropriate controls, replication, and sample sizes. The conclusions must be drawn appropriately based on the data presented.

Reviewer #1: Yes

Reviewer #2: Yes

Reviewer #3: Yes

---

2. Has the statistical analysis been performed appropriately and rigorously?

Reviewer #1: Yes

Reviewer #2: Yes

Reviewer #3: No

---

3. Have the authors made all data underlying the findings in their manuscript fully available?

The PLOS Data policy requires authors to make all data underlying the findings described in their manuscript fully available without restriction, with rare exception (please refer to the Data Availability Statement in the manuscript PDF file). The data should be provided as part of the manuscript or its supporting information, or deposited to a public repository. For example, in addition to summary statistics, the data points behind means, medians and variance measures should be available. If there are restrictions on publicly sharing data—e.g. participant privacy or use of data from a third party—those must be specified.

Reviewer #1: Yes

Reviewer #2: Yes

Reviewer #3: Yes

---

4. Is the manuscript presented in an intelligible fashion and written in standard English?

PLOS ONE does not copyedit accepted manuscripts, so the language in submitted articles must be clear, correct, and unambiguous. Any typographical or grammatical errors should be corrected at revision, so please note any specific errors here.

Reviewer #1: Yes

Reviewer #2: Yes

Reviewer #3: Yes

---

5. Review Comments to the Author

Please use the space provided to explain your answers to the questions above. You may also include additional comments for the author, including concerns about dual publication, research ethics, or publication ethics. (Please upload your review as an attachment if it exceeds 20,000 characters)

Reviewer #1: This article is simple in concept and design but the authors thoroughly and elegantly describe the justification of their work and the results. I have minor comments that I think should be addressed in the discussion section.

1) The authors state that preference tests were performed comparing items from different "categories" of enrichment, but all the comparisons presented show preferences within categories. Did the mice have consistently any preference for one category over another? This could actually be worth making a figure ranking the categories in each group to visualize any effects (or lack thereof).

Thank you for this interesting consideration. However, an overall comparison of enrichment items from different categories is not feasible with our obtained dataset. With an increasing number of items the number of binary comparisons increases according to the formular (number of items-1)*(number of items/2). Overall we included 15 items in 3 categories in our comparisons. If we had included all possible combinations, overall (15-1)*15/2=105 instead of 30 experiments had to be done. In addition, we would expect a clear preference for a cage where a housing enrichment was placed if no housing but an element of the foraging category was placed in the other cage which would lead to unfair comparisons. We therefore deliberately chose to do the comparisons within the respective categories only to reduce the number of comparisons and to come to results that are more meaningful. In the revised version we elaborate on this in more detail (lines 444 ff. and 496 ff.).

2) This study was performed exclusively in female mice. The authors cite an article investigating enrichment in male mice, but do not compare their results in the discussion section. With the importance of sex as a biological variable I think this is worth discussing, especially since I cannot seem to find access to the cited article studying male mice.

We assume that you are referring to the study on work motivation for enrichment by Lewejohann and Sachser, 2000 (it's a little bit hidden but available here: https://www.ktbl.de/fileadmin/user_upload/Allgemeines/Download/DVG-Tagung/P_11391_small.pdf). In that study tests with male mice confirmed previous results found in female mice.

We agree that considering sex as a factor in the study of preferences for enrichments in mice is very important. According to the suggestion, we have included this point in our discussion (LL 215 ff).

3) It may be worth pointing out the age of the mice in the discussion section. These mice are about a year old, which I would consider to be significantly older than the age of subjects used in most studies. How might these results compare to mice closer to a more common experimental age (e.g. 2-4 months old)? Could this question of enrichment quality, selection, and variation be of particular relevance to studies making use of older animals (e.g. aging studies, Alzheimer's research)?

Although unfortunately literature on this subject is sparse, we added this topic to our discussion (LL 359 ff) because we see it as an interesting area for future preference studies.

Reviewer #2: The manuscript of Hobbiesiefken et al. examines how mice housed a group setting show individual preferences for particular structural, housing, and foraging enrichment objects. Using ID tagging of each mouse in combination with a two connected home-cage based assay, they track the preferences of individual mice over a 46 hour period and show that individual mice as well as groups show varying preferences for objects with no clear preference across the whole cohort. On a whole, I thought the study addresses a useful consideration in behavioral neuroscience, was interesting to read, and does a good job making use of ID tagging technologies to examine how mice influence each other's behavior in the homecage.

I have a few questions and suggestions which I feel would strengthen the conclusions of the manuscript:

1. My main concern is that the data about individual animal preferences may be heavily confounded by time spent in one object's chamber but not directly interacting with the object. As the authors noted, time spent on one side or the other can be biased by the presence of a nest, for example, where the animals may choose to sleep or rest for long durations of time. Morever, some animals may innately be less exploratory than others, and may spend more time in the periphery of the cage rearing or resting even when awake. As such, I think the authors ought to analyze the data in a more precise manner by using the tracking data to identify bouts when the animals are directly interacting with the object, for example by thresholding for frame in which the animal is within a certain distance of the object. The authors could also complement their RFID tracking with multi-animal pose estimation to do this if necessary, as I'm not totally sure if RFID will provide the spatial resolution for this. If the video quality is good enough, it would be doable to implement multi-animal Deep Lab Cut or SLEAP (www.sleap.ai) for multi-animal pose tracking. SLEAP in particular is very user friendly.
Thank you for your suggestions for further analyses of preferences for enrichment items in mice. We based our enrichment study on previous methods for assessing preferences in mice, such as those used by Blom et al. (1992) to calculate dwelling times in differently equipped cages. We agree that a parallel calculation of time spent directly with the enrichment items would be useful. However, the spatial resolution of our RFID setup enabled calculation solely of dwelling time in either of the two cages. By subdividing the results into the dark (active) phase and the light (inactive) phase (ll 235 ff) we further took into account the influence of time spent supposingly resting in a nest or actively engaging with the enrichment. We discuss this especially related to the different categories of enrichment i.e., serving for active engagements and for housing enrichments used for resting. The possible influence of the nesting side on preference behavior was also mentioned as a limitation in our discussion (ll 208 ff). A video observation of direct interaction with the same enrichment items was conducted prior to this study and published in 2021 (Hobbiesiefken et al., 2021). Here we focused exclusively on the calculation of dwelling time in order to provide an automated measure. Recent methods for automated evaluation of behavior by deep learning algorithms using video observations such as Deep Lab Cut or SLEAP are indeed promising approaches to gather additional information for future studies.

2. I thought that categorizing behaviors as "influencing" vs "following" events was very interesting and was curious if individual mice are influenced by particular other mice more than others? i.e. maybe animal 1 is highly influenced by animal 2, but animal 3 is more influenced by animal 4? Or is there one mouse that clearly influences the other 3 in a more

generalized manner? Either way regardless of the outcome it would be very interesting to show the data.

We analyzed the data with regard to who is influencing whom and included the results to the supplements (S1 Fig). In brief, there is no prominent pattern indicating that any of the mice was leading or hindering its conspecifics.

3. The data set is quite a rich one due to the sheer length of time that the behavior was recorded for. It would greatly enrich the manuscript to show how preferences evolve (or remain stable?) across hours and different phases of the light-dark cycle. In the manuscript the authors wrote that they analyzed this, but do not seem to state the results or show the data. Unless I am missing something?

We observed the preference over two active and two inactive phases with changing the side of the presented elements in between. Due to the high number of item combinations we refrain from analysing the temporal development of preferences. However, we compared each set of enrichments during the active and inactive phase and the overall testing period of 46 hours of the behavioral analysis. The data can be found in Fig 1..

4. Did the authors rank the mice? If so, is there any correlation between social rank and preference for objects? Or any correlation between social rank and percent time influencing vs following? (If they did not rank the mice, I am not suggesting that they re-do the experiments with ranking).

We did not determine a rank order of the female mice, and overall agonistic interactions are very scarce in group housed female C57Bl/6. Nevertheless, we admit that it is not unlikely that the groups established subtle rank orders. The newly added analysis of how the mice mutually influence each other, however, indicates that no clear pattern emerged which would suggest an influence of a potential rank order.

Reviewer #3: Hobbiesiefken et al. address the critical question of what types of housing enrichment socially-housed laboratory mice engage with most. They name specific items with the highest worth values, but find substantial variation in preference among individuals and groups tested. This work has important implications for how mouse researchers and animal technicians care for their animals, which the authors give strong recommendations for in closing their discussion.

The statistical methods used appear sound given the researchers' choices, but I see several alternate choices that would provide new opportunities for analyses that would round out the story or make the findings more behaviorally relevant. These recommendations are included in several comments below.

I appreciate the authors' assertion that elements with low worth values or high consensus error may still be enriching for mice (line 251). An additional consideration is whether there was an aversion to any item. Does a low worth value indicate an aversion? What would be a threshold or difference to determine an aversion for any particular elements? Are statistical comparisons possible with the worth value data?

The worth values indicate the probability that a certain element is preferred over the other items based on all possible comparisons. It would also be possible to include aversive items in such a ranking, and if only one clearly aversive item was included we would expect that this item has a worth value of 0 (and also a CE of 0). However, if a number of aversive items was ranked, the least aversive item would receive the highest (non-zero) worth value. Thus, a worth plot on its own can only indicate the relative order but not the true valence of the tested items. Nevertheless, from direct observations (Hobbiesiefken et al., 2021) we know that mice interacted with all enrichment items and none of the items was avoided. Therefore we consider an item with a lower worth value as just being less attractive rather than avoided.

One consideration that the authors have nearly overlooked is the different dynamics of socially-housed mice compared to isolated mice. While social housing is and should be the norm, there are many conditions in which mice are housed individually (i.e. for research or medical purposes). Enrichment is arguably even more important for individually-housed mice, and preferences may be different under those circumstances. This should be addressed in the manuscript beyond explaining that singly housed animals were not used; I believe this warrants separate experiments, but at the very least this should be acknowledged in the discussion.

We agree with you that enrichment of the housing of singly housed mice is even more important than for group housed mice that at least have the group dynamics as a form of social enrichment. In the discussion we now elaborate on possible differences between single and group housed mice and added the suggestion that enrichment for single housed mice might be even more important (ll 223 ff).

I have several issues with the analysis of influence within a cage.
First, there is an assumption that social influence would only positively influence the preferences of enrichment items – only "follows" are assessed. It is likely that there could be an effect in the opposite direction: one mouse interacting with an item could lead to another mouse choosing a different item – for example, due to avoidance of the cagemate, or to some logistical ease of playing with a separate toy.
Second, and related to the first, just looking at "follow" events is very limiting. A more relevant metric both to address influence and how mice engage with their items would be how much time they spend with at least one other mouse using the item at the same time.
Third, both the 1 and 3 second follow criteria are arbitrary and don't have any rationale. Imagining a "follow" in under 1 second, I see one mouse blindly following another, practically in lock-step, regardless of what it was doing, which doesn't seem natural. If instead I imagine a scenario where a "follow" seems natural, I imagine Mouse 1 moving to a new cage/enrichment object while Mouse 2 is doing its own thing, then notices 1 is gone and/or playing with a new object, then decides to follow, then stops its own activity, then follows. This could take many more than 3 seconds and still be socially relevant. See second point above for one alternative analysis, but there are others including expanding the time for analysis that I think would be more relevant.

We admit that the timing of 1 and 3 seconds we used to analyze follow events is arbitrary. In contrast to the scenario the reviewer described, our approach using a threshold is measurable and indeed following events with one mouse directly lining up to another conspecific are relatively common. Nevertheless, we now added an additional more elaborate analysis (S1 Fig) showing the time spent together for any of the possible dyadic combinations within each cage. This data does not at all indicate that mice avoid each other in any diadic combination.

I don't understand how the statement "This supports the hypothesis that nest boxes are also perceived as important exploration objects for mice rather than a mere refuge and sleeping area" (line 298-300) follows from the preceding finding that mice had no real preference of housing enrichment during their inactive phase. It seems this shows instead that regardless of how a housing element is used during the active period, they will all be similarly used during the inactive period.

Thank you very much for your comment. We have indeed expressed ourselves in an incomprehensible way and have adjusted the sentence in accordance with your comment. It now reads: 'This suggests that even though nest boxes are perceived as important exploratory objects for mice during the active phase rather than just a mere refuge and sleeping area there is little difference in use during the inactive period.'

Line 315 – what are consumer demand tests?

Consumer demand tests in animals are used to determine the relative strength of motivation to obtain resources, e.g. food. As an example this can be achieved by training the animals to press a lever, to gain access to certain goods. By slowly increasing the "price" in the form of lever presses, one can compare the value of different resources from an animal's point of view. We added additional information about consumer demand tests in our discussion (ll 353 ff).

A final important consideration for advising a huge change in housing conditions is how that may affect the mice (e.g. their brains) and how that could influence future research conducted with "enriched" mice, especially for comparison with past research.

It is known from several studies that housing conditions indeed affect the behavior as well as the brain of mice. While this historically has been discussed in terms of enrichment changing the animals, newer literature coins it the opposite way pointing out that impoverished housing has detrimental effects. We share this view and emphasized this point more clearly in the introduction (ll 63 ff) and discussion (ll 376 ff).

Figs 1-2: What is the significance of the line connecting the items on the graph? The size of the dots? I think it would be helpful to see all the graphs have the same Y-axis range, and to have all of the item names be arranged in a single column rather than scattered, which makes the graph look like it is more dimensional than it is.

We tried different scalings of the y-axis for the readable display of the results from the preference tests. As you may have noticed, the results in certain categories are so close together that a legible representation with a constant y-axis scaling was unfortunately not possible. In order to increase readability, we decided to display the values with different scaling between the categories, but still keep the same scaling within a category. The vertical lines are part of the plot and indicate that the respective items belong to a group for which the values are calculated in dependence on each other.
